# Locomotor Ataxia

**Published:** 1883-01

**Authors:** A. M. Carpenter

**Affiliations:** Professor Practice of Medicine, College of Physicians and Surgeons, Chicago


### ARTCILE III.

LOCOMOTOR ATAXIA. CASE. RECOVERY. By A. M. CARPENTER, M. D., Professor Practice of Medicine, College of Physicians and Surgeons, Chicago.

It is so unusual for cases of the ataxic type to eventuate in perfect restitution, I feel constrained to lay before your readers the history of a well-marked instance—which may not correspond accurately with the description given of the affection by recognized authorities, but nevertheless quite prominent in its essential features.

November 13, 1879, was asked to visit F. W., aged 21, and treat him for paralysis. Coming into his presence, he at at once apologized for his appearance physically, by stating that he was not intoxicated, as some of his neighbors had supposed, because of his tottering gait.

When asked to exhibit his walk he consented, staggering across the apartment, aided by two canes—declaring he had never drunk. The gait disproved the existence of paralysis; the heel struck hard after a spasmodic effort; the ball of the foot followed after an interval; no arc of a circle or dragging was described. Nor had he the faculty of adapting either foot to any special point. The same want of co-ordination obtained in the finger, which would alight on the chin instead of the lip; the distance between the feet during attempts at locomotion was diagnostic, especially while standing, which he could not do with closed eyes—nor was he capable of standing to do his morning ablutions without toppling over. The inability to walk increased, until he had to be supported. Anæsthesia of the plantar aspect was present. Sensory impressions were slowly conveyed, as evidenced by the prick of a needle not being felt for several seconds, while a heated iron was discernible, and responsive movements of the muscles was observed when tickling was practiced, which, according to writers, is explicable on the ground of there being four sets of fibers resident in each sensiferous nerve—and though these multiple fibers are in close companionship, they are susceptible of functionating activity as individual nerves, and may be pathologically deranged in their separate capacities without the nerve being altered in its entirety. Applying a hot sponge to the skin produced no effect, nor was there present "the constricting band" sensation so frequently present in myelitis, but our patient was sorely harassed with neuralgic pains, which were not domiciled in one spot, shifting from lower to upper extremities.

There was more or less confusion of intellect. The bladder was under the volition of the patient, which is not the case in paraplegia. Seeing that we had to wrestle with ataxia, the next point of inquiry was as to the prominent factor which sustained a causative relation to the malady. It was ascertained that he had suffered an attack of malarial fever, of a remittent type, some weeks prior to my visit, but which was regarded as insufficient of itself to have produced such a train of consequences. Upon further search, the discovery was made that the youth was an inordinate user of tobacco, and of the virile member in a solitary way—being of continent habits had never had congress with a female. The conclusion was reached that the lumbar spine had sustained the greatest shock, in acccordance with which our judgement was delivered, and hope of recovery held out to the family of the sufferer because of the age of the patient, and his general good habits aside from those already mentioned. Absolute quiet was enjoined as one of the conditions of success. The curious were prohibited seeing him, and shortly his tongue lost its confusion. In the course of a few weeks electric sensibility was restored to the extremities through the aid of spinal galvanization and general faradization. The hæmatosic function, which had been embarrassed since the attack of remittent, was gradually restored under the use of twenty-drop doses of dialysed iron, thrice daily. Ergot signally failed to better the condition of the cord, when the sixtieth of a gr. of phosphorus in union with the thirtieth gr. of strychnia, thrice daily, was employed for ninety days, with general amendment in all the symptoms, at which time he could propel himself with two canes very comfortably, though the gait was not quite normal. After the lapse of six months from the date of the attack locomotion was perfect. A letter of date of September last, from the patient, states that there has been no return of malady or anything akin to it.

It is foreign to the object of this hurried report to offer the plan of management as containing anything new. Suffice that it was a fortunate case for experimentation with stereotyped measures. It is possible that the diagnosis was faulty, and that the disease was an incubative state betweeen congestion and induration, which may be a crumb to those who aver that sclerosed cords are never recovered from. Whether rated as functional or lesional in its type the fact stands out, that according to the history as portrayed by some neuropathologists, it was a well-declared case of ataxia; was so adjudged, and perfectly recovered under a recognized plan of management with which every observant medical man is familiar.

